# Differential assessment of fluid compartments by bioimpedance in pediatric patients with kidney diseases

**DOI:** 10.1007/s00467-020-04912-w

**Published:** 2021-02-12

**Authors:** Sandra M. Frey, Bruno Vogt, Giacomo D. Simonetti, Rainer Büscher, Sandra Habbig, Franz Schaefer

**Affiliations:** 1 Department of Nephrology and Hypertension, Inselspital, University of Berne, Bern, Switzerland; 2 Medizinische Klinik, Spital Region Oberaargau, SRO AG, Spital Langenthal, St. Urbanstrasse 67, 4900 Langenthal, Switzerland; 3 Pediatric Institute of Southern Switzerland, Ente ospedaliero cantonale, Bellinzona Università della Svizzera Italiana, Lugano, Switzerland; 4 Department of Pediatric Nephrology, University Hospital Essen, Essen, Germany; 5 Department of Pediatric Nephrology, University Children’s and Adolescent’s Hospital, Cologne, Germany; 6 Pediatric Nephrology Division, Center for Pediatrics and Adolescent Medicine, University of Heidelberg, Heidelberg, Germany

**Keywords:** Chronic kidney disease, Whole-body bioimpedance spectroscopy, Children, Extracellular fluid volume, Intracellular fluid volume, Hydration status

## Abstract

**Background:**

The kidney is central for maintaining water balance. As a corollary, patients with impaired kidney function are prone to pathological fluid volumes. Total body water (TBW) is distributed between the extracellular (ECW) and intracellular fluid compartments (ICW). In clinical practice, the judgment of hydration status does not allow to distinguish between ECW and ICW. Here, we evaluate the hydration status in children with chronic kidney disease by analyzing TBW, ECW, and ICW.

**Methods:**

Hydration was quantified using whole-body bioimpedance spectroscopy (BCM) in 128 outpatients (1–25 years, 52 girls). Forty-two were transplanted (TPL), 43 suffered from chronic kidney disease without kidney replacement therapy (CKD), 21 were on peritoneal dialysis (PD), and 22 on hemodialysis (HD). HD patients were investigated before, after, and sequentially during dialysis.

**Results:**

The ECW and ICW values obtained by BCM were of the same magnitude as those from the literature using isotope dilution. When compared with a healthy control group, TBW was increased in 9 TPL, 9 CKD, 1 PD, and 11 HD patients before but in none after dialysis. The decline of overhydration during dialysis (*p* < 0.001, *n* = 22) correlated with the change in body weight (*R*^2^ = 0.62). The kinetics of fluid compartment changes assessed twice in six HD patients revealed a reproducible linear decay of the ECW/ICW ratio due to an increase of ICW and a decrease of ECW.

**Conclusion:**

BCM quantifies TBW and acute changes of ECW and ICW in children with chronic kidney failure. The clinical utility of measuring TBW, ECW, and ICW should be defined in the future.

## Introduction

Patients with impaired kidney function have a high risk of chronic hypervolemia [1]. Chronic hypervolemia induces hypertension and increased left ventricular mass [2–4]. Thus, and not surprisingly, cardiovascular diseases are an important cause of morbidity and mortality in patients with chronic kidney failure [4]. Precise recognition of the fluid status is therefore of practical importance in kidney patients. Fluid management is especially relevant in pediatric patients, because of the frequently quick changes of fluid volumes [5]. The estimation of “dry weight” in a constantly growing organism is one of the most difficult challenges for the pediatric nephrologist [6]. For quantifying such quick changes, the methodology for measuring fluid status has to be noninvasive and versatile. Until recently, the judgment of hydration status was by and large based on subjective clinical assessments, as no objective, easy-to-handle, and precise tools for measuring fluid compartments were available [5, 7]. Furthermore, the clinical assessment of fluid status does not allow distinguishing between intracellular and extracellular fluid volumes.

For several years, novel technologies have been in process to be developed for the quantitative assessment of the fluid status in humans. Among these techniques, whole-body, single- or multi-frequency bioimpedance spectroscopy are versatile noninvasive approaches for obtaining instant information about the body composition [8]. As indicated by Jaffrin et al., single frequency current will not penetrate completely into the cells and therefore does not measure the entire ICW [9]. A better distinction between ECW and ICW appears to be obtained by multifrequency bioimpedance spectroscopy, the method used in the present investigation, because both the ECW and ICW might be modulated in patients with kidney disease [9]. The calculations used for the body composition monitor (BCM, Fresenius Medical Care, Bad Homburg, Germany) are based on a physiologic tissue model [10]. The BCM was well-validated by invasive gold standard dilution methods using deuterium or tritium for total body water (TBW), bromide for extracellular water (ECW), and total body potassium for intracellular water (ICW) [10]. Recently, Dasgupta et al. validated the BCM in children for fluid status considering deuterium concentration decline and Urea Kinetic Modeling for comparison [5] and Eng et al. performed a longitudinal study comparing the BCM with the clinical judgments of volume status as assessed by peripheral and central aortic blood pressure as well as echocardiography and natriuretic peptide [11]. Both studies concluded that the BCM is an objective tool to define the hydration status in children.

In the present investigation, the fluid status was quantified by the BCM in an unselected outpatient cohort of 128 children with kidney failure with and without kidney replacement therapy. Furthermore, the acute kinetics of the changes of the extracellular to the intracellular fluid volumes as a function of fluid removal during hemodialysis treatment was investigated.

## Methods

### Patients

The present study was conducted at the Division of Nephrology of the Centre for Child and Adolescent Medicine, University of Heidelberg, Germany, in cooperation with the Departments of Pediatric Nephrology at the University Hospitals of Cologne and Essen, Germany. One hundred and forty-one Caucasian outpatients, aged 1‑25 years, with chronic kidney failure participated in the present study. One hundred and twenty-eight children and adolescents were included in the analysis, while 13 were excluded, as no reliable bioimpedance measurements could be performed due to restlessness and agitation (9), spasticity (3), or an unphysiological result (1). The final cohort was composed of 22 patients on hemodialysis (HD), 21 treated with peritoneal dialysis (PD), 42 with a kidney transplant (TPL), and 43 suffering from chronic kidney disease without kidney replacement therapy (CKD). Subject characteristics are given in Tables 1 and 2. The stage of chronic kidney disease (G1/G2/G3/G4/G5), were 4%, 13%, 26%, 46%, 11%, and 16% in CKD and 32%, 47%, 5%, 0% in TPL.

**Table 1:** Subject characteristics stratified by gender [median (range)]

	TPL		CKD		PD		HD	
	Girls	Boys	Girls	Boys	Girls	Boys	Girls	Boys
*n*	15	27	21	22	7	14	9	13
Age (years)	15	14	14	14	13	11	16	15
	(3–21)	(7–25)	(2–20)	(2–18)	(3–17)	(3–25)	(11–19)	(9–23)
Height-for-age (years)	13	11	12	12.5	12	9	13	12
	(4–18)	(6‑20)	(1–19)	(2–18)	(1–18)	(2–17)	(5–18)	(7–18)
Height (m)	1.6	1.5	1.6	1.5	1.5	1.3	1.5	1.5
	(1–1.7)	(1.2–1.8)	(0.8–1.7)	(0.9–1.8)	(0.7–1.7)	(0.9–1.8)	(1.1–1.7)	(1.2–1.8)
BMI (kg/m^2^)	20.1	18.9	17.1	18.2	17.6	16.7	19.7	17.9
	(14–27.3)	(12.8–28.1)	(12.6–24.4)	(13.1–25.4)	(11.9–23.1)	(13.3–30.7)	(14.4–33)	(12.8–26.4)

*TPL*, patients with a kidney transplant; *CKD*, patients suffering from chronic kidney disease without kidney replacement therapy; *PD*, patients on peritoneal dialysis; *HD*, patients on hemodialysis treatment

**Table 2:** Extracellular and intracellular fluid status, Blood pressure, GFR, Kt/V [median (range)]

	TPL	CKD	PD	HD before	HD after
*n*	42	43	21	22	22
TBW/kg	0.55 (0.37–0.67)	0.58 (0.24–0.79)	0.59 (0.44–0.71)	0.60 (0.38–0.69)	0.59 (0.38–0.69)
ECW/kg	0.24 (0.18–0.28)	0.25 (0.20–0.29)	0.24 (0.20–0.30)	0.26 (0.18–0.31)	0.23 (0.18–0.30)
ICW/kg	0.30 (0.19–0.40)	0.33 (0.22–0.49)	0.35 (0.23–0.43)	0.33 (0.20–0.40)	0.35 (0.20–0.43)
(ECW/kg)/(ICW/kg)	0.80 (0.66–1.04)	0.76 (0.66–1.22)	0.75 (0.58–0.93)	0.78 (0.66–0.92)	0.65 (0.56–0.93)
Diastolic BP (mmHg)	67 (50–90)	68 (44–101)	70 (42–100)	78 (53–118)	73 (37–118)
Systolic BP (mmHg)	121 (93–143)	113 (83–160)	114 (76–150)	134 (101–178)	116 (95–168)
MAP (mmHg)	86 (71–102)	84 (58–113)	86 (53–117)	96 (73–134)	85 (60–132)
GFR (ml/min*(1.73m^2^))	56 (26–140)	26 (5–116)	–	–	–
Kt/V	–	–	–	–	1.7 (1.2–3.0)

*TBW/kg* total body water; *ECW/kg* extracellular water/kg; *ICW/kg* intracellular water; (*ECW/kg)/(ICW/kg*) ratio of ECW/kg to ICW/kg; *BP* blood pressure; *MAP* mean arterial pressure; *GFR* glomerular filtration rate; *K* dialyzer clearance of urea; *t* dialysis time; *V* volume of distribution of urea

### Body composition monitor

The body composition monitor device (BCM), based on whole-body bioimpedance spectroscopy, is a tool to collect noninvasive information about body composition. The analyses were performed with the tetrapolar BCM device (Fresenius Medical Care, Bad Homburg, Germany), using an alternating current from 50‑800 μA. Impedance was measured at 50 different frequencies between 5 kHz and 1 MHz and is composed of two parameters, resistance and reactance. The resistance (R, Ohm) is inversely related to the content of tissue water, and the reactance (Xc, Ohm), is directly related to the cell mass of tissue, i.e., cell wall capacitance [12, 13]. To evaluate TBW, ECW and ICW by the fluid volume model, the BCM defines extracellular and total body resistance by the Cole-Cole equation [1, 14, 15]. As recommended by Fresenius Company, a quality indicator cutoff value (Cole fit) of ≥ 70% was used. All data presented were derived from these measurements and compared with those of a normal healthy reference group consisting of 300 girls and 307 boys established by collaborators of the Fresenius Company and integrated into the software of the BCM [16].

### Data collection

After lying down for at least 5 min, the BCM monitoring was performed on all CKD, PD, HD, and TPL patients in a supine position with arms and legs spread apart from the body in accordance with the manufacturer’s manual (Fresenius, BCM operating instructions) [8, 16]. Four electrodes were attached on the back of one hand and the ipsilateral foot. The two proximal electrodes were placed on the wrist and ankle, and the other two electrodes were located at 3 cm distal of the proximal electrodes. In patients with a fistula, the hand without a fistula was considered. Patients with extremely small hands and feet had the electrodes positioned as distal as possible, not comprising fingers or toes. BCM measurements were performed before and after dialysis treatment in 22 patients. In six dialysis patients, measurements were performed on two dialysis sessions before and during dialysis (three to four measurements) and 20‑30 min after dialysis was stopped. In one dialysis patient, BCM was assessed 32 times before dialysis for a period of 6 months. In patients with peritoneal dialysis, the body weight considered was without any peritoneal fluid. The same operator (SMF) made all BCM measurements.

### Statistical analysis

The median and range are given. The Kolmogorov-Smirnov test and the Shapiro-Wilk test were done to control for normal distribution of the values. For multiple comparisons, ANOVA and paired *t* tests were applied. *R*^2^ is given when correlations were calculated. A *p* value of < 0.05 was considered to indicate significance.

## Results

**Fig. 1:**
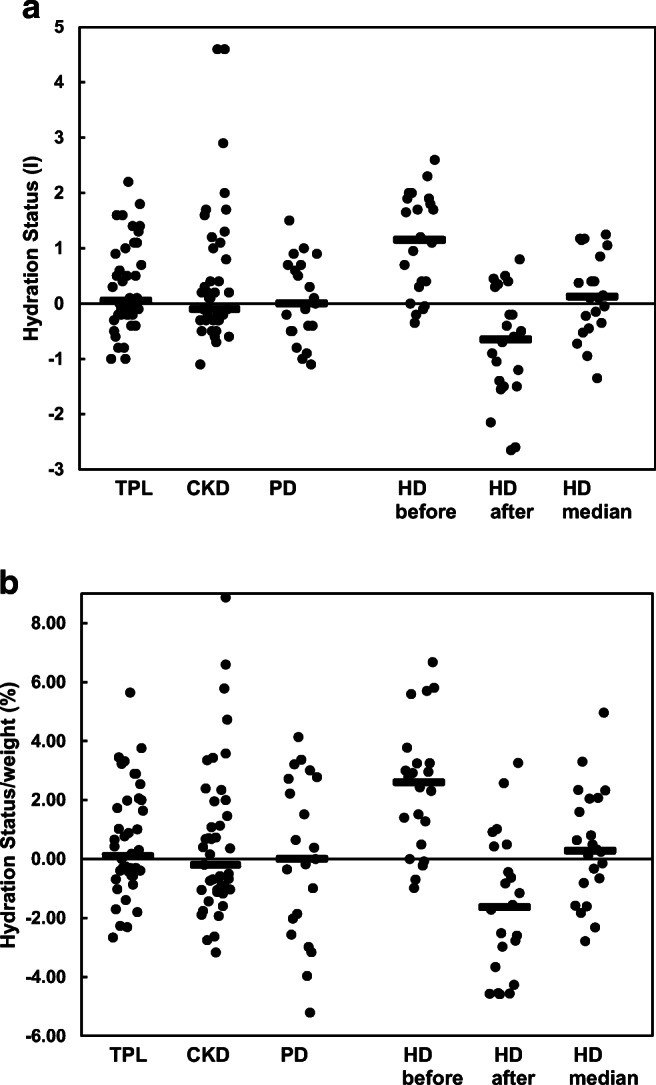
**a** Hydration status in liter in kidney transplant patients (TPL, *n* = 42), patients suffering from chronic kidney disease without kidney replacement therapy (CKD, *n* = 43), patients treated with peritoneal dialysis (PD, *n* = 21), and patients on hemodialysis (HD, *n* = 22). For the HD patients, the values before and after dialysis as well as the median from the value before and after HD session are given. Each dot represents the value of an individual patient. The black lines express the median value of the population. A positive value indicates a total body water volume-plus compared to the zero-reference line of the healthy population. When the values before HD with those after HD were compared, a significant decline was observed (*p* < 0.001). **b** Hydration status expressed as percentage of the body weight in kidney transplant patients (TPL, *n* = 42), patients suffering from chronic kidney disease without kidney replacement therapy (CKD, *n* = 43), patients treated with peritoneal dialysis (PD, *n* = 21) and patients on hemodialysis (HD, *n* = 22). For the HD patients, the values before and after dialysis as well as the median from the value before and after HD session are given. Each dot represents the value of an individual patient. The black lines express the median value of the population. A positive value indicates a total body water volume-plus compared to the zero-reference line of the healthy population. When the values before HD with those after HD were compared, a significant decline was observed (*p* < 0.001)

The patients were divided into four groups—patients with a kidney transplant (TPL), patients suffering from chronic kidney disease without kidney replacement therapy (CKD), patients on peritoneal dialysis (PD), and patients on hemodialysis treatment (HD) (Figs. 1a, b). Tables 1 and 2 show the subject characteristics as head count, age, height for age, height, body mass index (BMI), GFR, Kt/V, and arterial blood pressure stratified by gender with median and range of the 128 pediatric patients with chronic kidney disease investigated. In all groups of patients studied, the height-for-age was substantially lower than one would predict on the height of healthy children.

### Hydration status

The hydration status was measured with the BCM. In HD patients, measurements were obtained before and after hemodialysis sessions. In addition, from the two measurements, the median value was calculated for each HD patient. In Fig. 1a, the absolute value of the hydration status in liters (l) is given and in Fig. 1b, the relative fluid status is expressed as percentage (%) of the body weight. For each of the six groups, the median was calculated and added as a black line. The median value of the hydration status was close to the zero reference line reflecting normal hydration of the TPL, CKD, and PD populations. This was also true when the hydration status was expressed as percentage of body weight (Fig. 1b). No statistical differences were observed between the three groups. When compared with a healthy historic control group, TBW was increased in 9 TPL, 9 CKD, 1 PD, and 11 HD patients before but in none after dialysis. The dispersion of the results appears to increase when the absolute hydration status was expressed as percentage of body weight, especially in PD patients (Fig. 1b). This is probably due to the fact that some of the PD patients were very young (Table 1). No significant correlation between systolic BP, diastolic BP, mean arterial BP, GFR, stage of chronic kidney disease or Kt/V, and the hydrations status (ECW, ICW, OH) were found in the groups of patients studied.

**Fig. 2:**
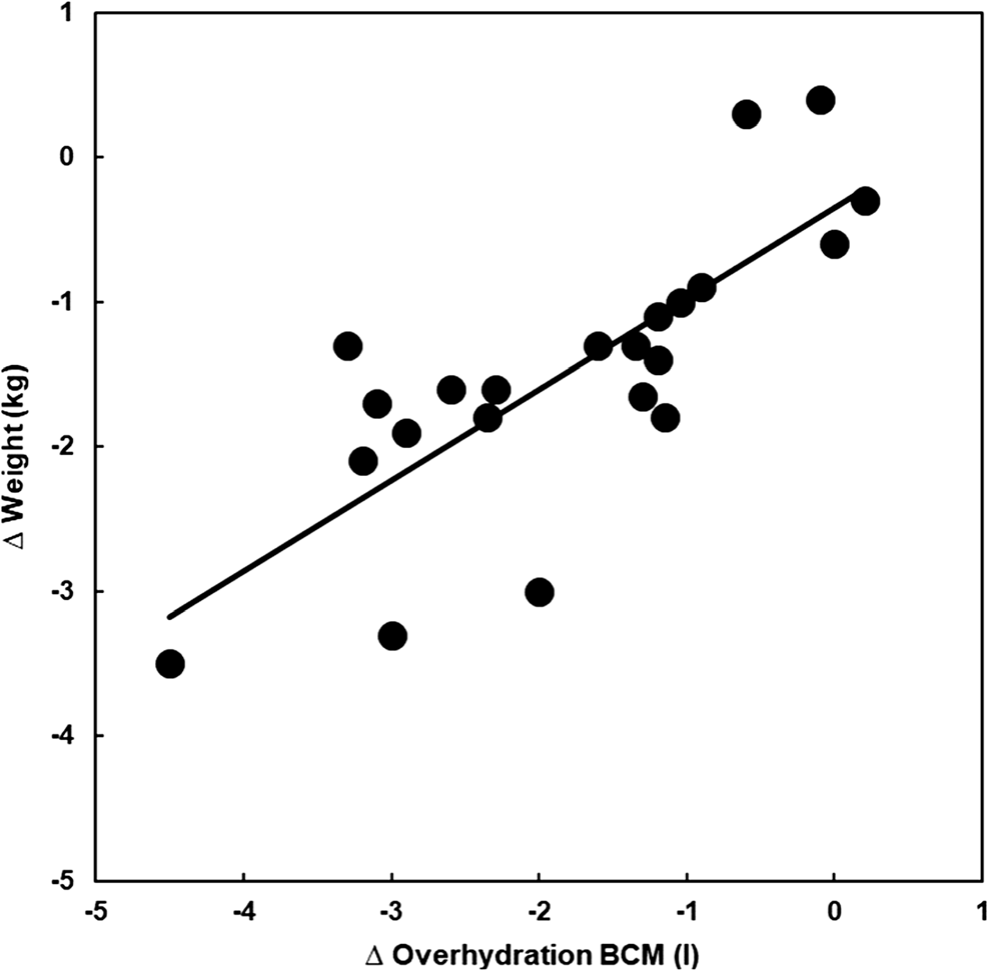
Changes of the hydration status as assessed by BCM in liter (*x* axis) vs. changes of the body weight measured by scale in kilogram (*y* axis) during a dialysis session. Each dot represents a patient. The two measurements appear to be interdependent: *y* = 0.63*x* − 0.35, *R*^2^ = 0.62, *p* < 0.001, *n* = 22

In hemodialysis patients, the absolute and relative fluid status increased before HD treatment and decreased after HD treatment when compared to normal values (Fig. 1a, b; *p* < 0.001). These values declined in 21 of 22 subjects and remained unchanged in one patient during HD (Figs. 1a, b and 2; *p* < 0.001). Interestingly, considering the median values derived from the measurements before and after HD treatment, an equal distribution with a median value close to the zero reference line was observed (Fig. 1a, b). The body weight (kg) was measured by a scale before and after HD treatment in all patients. The changes in body weight correlated with the changes in the hydration status as assessed by BCM (*R*^2^ = 0.62) (Fig. 2).

### Extracellular and intracellular fluid volumes

The BCM method was used to analyze extracellular (ECW) and intracellular (ICW) water per kilogram and the ratio of ECW/ICW was calculated (Table 2). The median and range of these parameters did not differ between TPL, CKD, and PD patients.

**Fig. 3:**
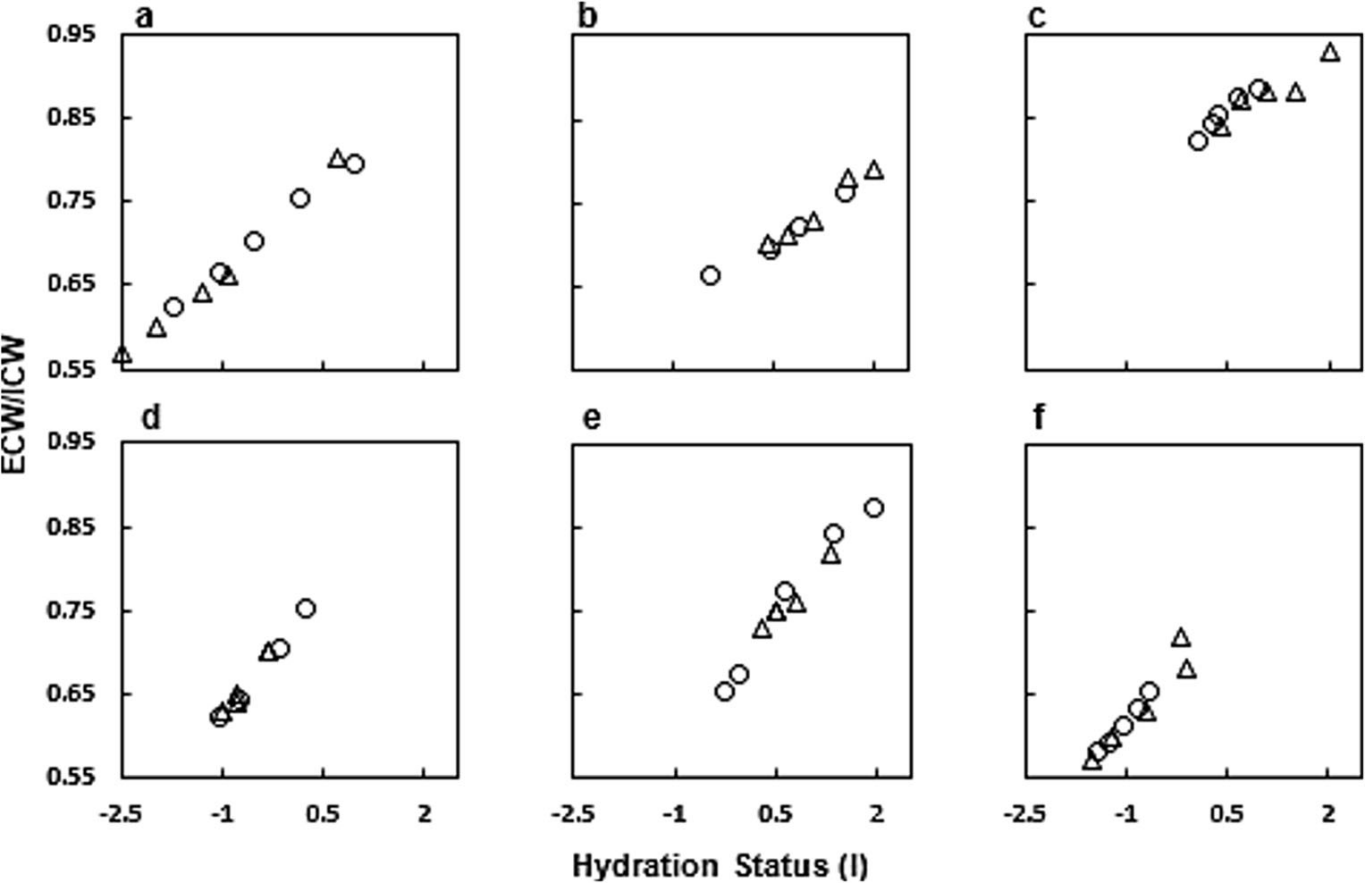
**a**‑**f** Changes in the extracellular to intracellular fluid volume (ECW/ICW) as a function of the hydration status (in liter) in six patients measured on two occasions during dialysis treatment by BCM. Open circles and open triangles represent the two dialysis sessions from one patient. In all patients investigated, the ECW/ICW ratio declined steadily when fluid was removed during dialysis. The decline during the two dialysis sessions parallels in every patient. The linear regression between the hydration status and the ECW/ICW was highly significant in all subjects, with *R*^2^ values ranging between 0.91 and 0.99, *p* < 0.001

**Fig. 4:**
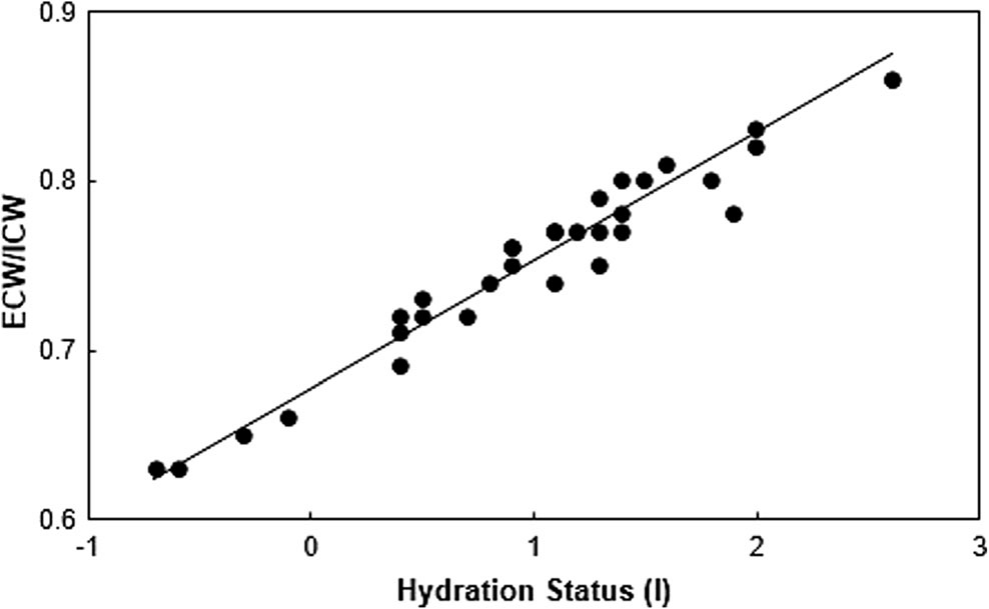
Changes in the extracellular to intracellular fluid volume (ECW/ICW) as a function of the hydration status (in liter) in one patient measured on 32 occasions before HD by the BCM during a period of 6 months. With declining hydration, the ECW/ICW volume ratio decreased: *y* = 0.08*x* + 0.68, *R*^2^ = 0.93, *p* < 0.001

In HD patients, the values of ECW declined in 19, those of ICW increased in 17 and the corresponding ECW/ICW ratio declined in 20 out of 22 patients (*p* < 0.001) (Table 2). In order to analyze the reproducibility of the results obtained by the BCM, two different approaches were designed. First, six patients were investigated on two hemodialysis sessions (Figs. 3a-f). The time elapsed between the two measurements was 9 days, with one exception of 1 day difference (Fig. 3d). As shown in the figures, the changes in the body water composition during the dialysis sessions were extraordinarily reproducible in all subjects (Fig. 3a‑f). Second, measurements were performed over a period of 6 months in one patient (Fig. 4). The patient was a 19-year-old girl with a BMI of 14‑15 kg/m^2^ who had a kidney transplant, which had to be removed because of transplant rejection before the measurements were started. The change in the hydration status was linearly linked with the changes of the extracellular to the intracellular volume, suggesting perfect intrapersonal reproducibility (Fig. 4).

## Discussion

The BCM used to perform the present study is based on whole-body multi-frequency bioimpedance. This device is a versatile noninvasive tool for gaining immediate information about the composition of the body. Even though it is a practical device, it has some limitations. First, the patient must keep still for 5‑15 s, which can be difficult to achieve when young children are investigated. In the present study, nine patients had to be excluded from the analysis because they could not keep still for the time required. Eight of these patients belonged to the group of 18 children of our cohort aged 3 years or below. Second, the results of one girl younger than 1 year were removed despite a sufficient quality indicator value of the method, because the derived result appeared to be unphysiological. Based on the observation that most children studied with an age below 2 years did not provide reliable results, one cannot recommend the method for assessing fluid compartments in children below the age of two. Third, we observed a previously unreported specific entity excluding reliable measurements with the BCM. Two patients on peritoneal dialysis and one patient on hemodialysis suffered from severe spastic disease. Interestingly, it was not possible to obtain reliable results for impedance from the BCM, which displayed a quality of 0%. The mechanism for the absence of qualitatively acceptable results is unknown, but we hypothesize that patients with spasticity might have a too high resistance in the limbs causing incorrect current flow. Fourth, for measuring bioimpedance, the electrodes are placed on one arm and one leg. Thus, patients with amputations cannot be investigated since alternative placements of the electrodes have to be performed and assumptions for the model used for calculating total body bioimpedance have to be considered [15, 17]. In our cohort, no patient had such a handicap. Fifth, measurements by the BCM might be modulated by artificial organs such as artificial joints, pacemaker, heart, or possibly others.

The weakness of the present investigation is the absence of a healthy control group for direct comparison. In order to estimate whether the present results are physiologically meaningful, we compared our results with those obtained from studies using gold standard methodology for assessing body fluid compartments, i.e., heavy water and bromide dilution. Planche et al. assessed body fluid compartments in 17 children aged 1 to 10 years 28 days after recovering from malaria in Gabon [18]. After recovery, their measured values of ECW (0.24 l/kg, median), ICW (0.31 l/kg, median) and the calculated ratio of ECW/ICW (0.77, median) compared well with the results obtained in the present investigation (Table 2), suggesting—serendipitously—absence of a substantial interethnic difference in the fluid compartments between Caucasian and African children. Buendia et al. analyzed 339 adults for defining body fluid composition using tracer dilution in a seminal study [19]. The ECW (0.22 l/kg, median) and ICW (0.26 l/kg, median) values obtained by Buendia et al. were slightly lower than those measured in the children by Planche et al. and in our study [18–20] (Table 2). This difference is not surprising since Edelman and Leibman summarized in 1959 that body water decreases progressively with age [21]. The median value of the ECW/ICW ratio in the adult group of Buendia et al. was 0.85, a value higher than that observed in all our patient groups and in the healthy children of Planche et al. [18]. The observed increase of the ECW/ICW ratio with age is due to a more pronounced decline in the ICW than in the ECW values [18–20] (Table 2). A similar age dependency on fluid volumes and their ratio were also identified by Fresenius Medical Care using the BCM. Thus, the absolute numerical values obtained in our study appear to be realistic.

Figure 1a depicts the hydration status expressed in liter in the different groups of our cohort. A positive value indicates a total body water volume-plus compared to the zero reference line of the population of healthy children composed of 307 boys and 300 girls given by Fresenius [16]. In this healthy children population, the hydration status changes with age. In the present investigation, we compared the results of our patients with those of the age-dependent reference values and considered the 10th and 90th percentile as the limit for normal hydration. Using this approach, nine subjects appeared to be overhydrated and one subject was underhydrated when the 42 patients with a kidney allograft were analyzed. The corresponding values were 1 and 1 for the 21 PD patients, whereas of the 43 CKD patients, nine were overhydrated and none underhydrated. Thus, the cohort of outpatients in the present study appears to be generally euhydrated. Interestingly, the largest fraction of subjects with an overhydration and underhydration was observed in the TPL cohort. It is conceivable that the impedance measurements might be modulated by the different body composition of kidney transplant patients as previously suggested based on CT measurements and morphometric analysis of microscopic ultrastructures [22, 23]. The low percentage of patients with overhydration or underhydration in the three populations discussed increases when the hydration status is expressed as a percentage of body weight (Fig. 1b) and not as liter per patient (Fig. 1a). This is best explained by the fact that some of the children are small for their age (Table 1) and their hydration status compared with the healthy children of the same age underestimates the abnormality of the hydration status.

During hemodialysis sessions, a substantial amount of fluid is removed from the body within a short time period. Thus, dialysis is an ideal experimental condition that allows testing the reliability of a novel method claiming to assess body fluid volumes. As shown in Fig. 1a and b, the hydration status declined in all patients during the hemodialysis session. When the patients were compared to the healthy control group mentioned above, 11 out of the 22 patients were overhydrated and none was underhydrated before hemodialysis treatment, whereas at the end of the dialysis session, none was over and nine were volume depleted. During the dialysis session, the intra-individual removal of fluid volume correlates with the change of hydration, indicating that the BCM results reflect physiological reality (Fig. 2). Hemodialysis removes fluid primarily from the intravascular space. Thus, it is relevant to analyze whether the acute removal of fluid changes the ECW and/or the ICW compartment. In all patients, a decrease of ECW and an increase of ICW were observed (Table 2). The increase of the ICW is best explained by the increase of the intra-/extracellular osmotic gradient due to the removal of urea by dialysis treatment [24].

As a corollary, the ratio (ECW/kg)/(ICW/kg) declined steadily and reproducibly in all six patients observed on two occasions during hemodialysis treatment (Fig. 3). The present observation of the changes in ECW/ICW is in line with a publication by Montgomery et al. [25]. These authors recently derived the intracellular and the extracellular fluid volumes in 17 kidney failure patients during hemodialysis by measuring tissue resistance and reactance of the calf [25]. As observed in our patients, the ECW volume decreased while that of the ICW increased. The absolute values of that investigation are not comparable with the values in the present study because Montgomery et al. assessed the impedance segmental on the calf. In line with our observation, the ratio of ECW/ICW diminished during hemodialysis treatment, although the absolute values were different. Using a technique similar to the present study, Jaffrin et al. analyzed the ECW and ICW obtained from seven adult patients three times before and after hemodialysis treatment [9]. They also observed a decrease of ECW and an increase of ICW. Using their average value to calculate the ratio of ECW/ICW the median result of pre HD was 1.20 and that of post HD 0.92. The ECW values expressed per kilogram body weight were of the same magnitude as those measured by Buendia et al. in healthy adult subjects. However, the ICW values were much lower, which explains their high ratio of ECW/ICW [9, 19, 20] (Table 2). The reason for the much lower ICW values observed by Jaffrin et al. is unknown. Thus, the noninvasive methodology allows assessing changes of extracellular and intracellular volume compartments during HD and the results appear to be intra-individually reproducible as shown in our patients (Figs. 3 and 4).

## Conclusion

The present study confirms that BCM can be used for measuring fluid status by determining TBW, ECW, and ICW in children with chronic kidney failure with and without the various kidney replacement modalities. The results obtained by BCM compared well with those from the literature using isotope dilution. BCM enables assessing acute extracellular and intracellular fluid shifts, is intra-individually reproducible, causes no side effects, and is easy to apply. Future studies should define the clinical utility of the differential assessment of ECW and ICW.

## Data Availability

Upon request.
